# Epsom College

**Published:** 1890-05-03

**Authors:** 


The Hospital

A Weekly Journal of

Science, Medicine, Nursing, and Philanthropy.

*For Hospitals, Asylums, Medical Practitioners, Students, Nurses, Families, Parishes, Congregations, and the Charitable Public.*

Vol. VIII.—No. 188.] [May 3, 1890.

### Epsom College.

What are called the "learned professions" are not very well paid in Great Britain, at least not in the minted coin of the realm. There are of course other rewards than money; rewards of much greater real and permanent value. The doctor shares with the clergyman, the soldier, and the statesman, the admiration and affection of his fellow men when his personal and professional merit are such as entitle him to that distinction. Without money the professional man is apt to be overlooked; but without esteem he cuts a very sorry figure. The law of supply and demand, as applied to doctors, has recently had the effect of cheapening their services, but it has not at the same time improved the value of those services. Skilled labour is generally well paid, and conversely, well paid labour is generally skilful. Reduce any man or body of men below a particular level of pay and you reduce, sometimes you even destroy, their capacity for work. A certain quantity and quality of food, a certain minimum period of sleep and rest, a certain measure of mental ease and freedom from wearing anxiety—these are indispensable to any and every man who is to do good work in the world. People tell us about the educational value of difficulties, tribulations, and the like; but such people are generally free from the suspicion of any difficulty or tribulation themselves. There is an educational value in prosperity as well as in adversity. Sunshine and fine weather must alternate with rain and cold or the world of life will make but a poor display.

Epsom College is a silent but true witness to the hardships and necessities of the modern medical man. It also bears testimony to the strength of the *esprit de corps*, and the intensity of the feeling of brotherhood that prevail throughout the ranks of medicine. That a good many medical men fail in the effort to make provision for old age is undoubted: that all those who fail honourably are regarded as having a just claim upon the sympathy and substantial help of their more prosperous fellows is proved by the existence and generous maintenance of Epsom College and its sister institutions. It is entirely good and right that medical men should help each other; it would be very much better if there were none who needed help. If medical services were paid for as they ought to be, there would be fewer necessitous old men and women of education and character to grieve the hearts of those who love justice. No man of high personal honour can accept help from his fellow-men without humiliation, nor can he sue for such help on behalf of his professional brethren without a blush. And yet, when men have fought the battle of life bravely, and have been wounded and reduced to want in the struggle, it ought to be considered that they are merely accepting the payment of a just debt when they allow themselves to receive pecuniary aid from others.

It is not necessary to explain to medical men the important services rendered by Epsom College to the rank and file of the profession, to the orphan children of those who have died before the battle of life was won, and to the aged who have outlived their capacity for self-maintenance. But for non-medical readers who may be conscious of obligations to particular physicians or surgeons, and through them to the whole medical profession, a word or two of explanation may be desirable. Epsom College is first and foremost a public school, where the sons of medical men and others may receive a liberal education at a moderate cost. There is combined with the school a "Benevolent" scheme whereby the orphan children of medical men who have died leaving inadequate provision for their families, may also have the full advantage of a public school education without any loss of social status or just self-regard. Instead of being sent to a purely charitable institution and thus being compelled to associate only with boys in a similar position, they become equal members of a self-maintaining educational commonwealth, and retain all the rights, privileges, and amenities which are common to the whole school. Not only are the orphan sons of medical men thus gently and considerately cared for, but so far as is possible a certain number of aged medical men and their wives, who are no longer able to work for their own maintenance, are provided with homes and small incomes which enable them to live in modest comfort and simple dignity for the few remaining years of their lives. Epsom, then, is an educational institution which spreads one sheltering wing over fatherless youth and the other over defenceless age. That being so, we cannot but feel that it has undoubted claims upon prosperous medical men, and may not improperly seek from outsiders a considerable measure of aid in its benevolent work.

The medical profession makes great efforts to keep its own necessities within its own borders. There are several schemes, of a provident as well as of a benevolent character, in existence which aim at securing provision both for age and helpless youth. Those institutions are highly valued and well supported. But they do not quite cover all the ground that needs to be covered. In spite of the best efforts there are still some, both aged men and women, and young children, who are driven by the stormy tide of life on to the rocky shores of absolute poverty. Epsom strives to rescue these, and we cannot but think that in so doing she may justly and without humiliation claim some sympathy and help from humanity at large, as well as from the medical profession. Nay, is it not the case that because the medical profession so nobly strives to provide for all its necessitous ones, and so nearly succeeds, therefore it should with all the more confidence and hope make known its need for aid in order that its work may be rendered quite complete? We can well understand why Epsom College should proudly refuse to beg for itself. But we can also understand why so many of those who see and know the good work it is doing by its benevolent department should wish to have a practical share in that work.

A week or two ago the College had its Biennial Festival Dinner under the presidency of Sir James Paget. There was a distinguished company present, and a liberal subscription list was the result. But a letter from the Treasurer, which we published last week, informed us that the list is to be kept open until the annual Governors' meeting, to be held on May 22nd. There is also, we believe, a large scheme on hand by which it is intended to raise £100,000. The object of this is to provide a capital sum, the interest of which shall be devoted exclusively to the providing of modest pensions for aged and infirm medical men and their wives. If this scheme is ultimately successful, as we cannot doubt it will be, medical pensioners will no longer require to live in what may be called "almshouses" at Epsom, but will be able to remain comfortably in the towns and villages where their active lives have been spent. The Epsom "Pensioners'" houses, which are greatly needed for the extension of the school, will thus be set free for that purpose. In this scheme an opportunity is offered to those who have money and who sympathize with or are grateful to the medical profession, to show their appreciation by their deeds. We shall not be misunderstood when we say that doctors deserve well of the public. Nineteen-twentieths of them work as hard as any slaves, but they seldom secure either riches or public honours. They do their duty from day to day; they earn the confidence and friendship of their patients; and they certainly fill a place in modern life and activity which, if left vacant, would result in irreparable injury to civilization and human progress. On the whole, the medical profession is not valued according to its deserts. The world owes it very considerable arrears, both of wealth and honour. The science of the times, which is in a large sense the material salvation of the times, is, to a very great extent, the product and offspring of medicine and the allied professions. The world does not know this apparently; and cares as little as it knows. That is not quite as it should be. Generous minds love to make reparation. We suggest to our non-medical readers that they might take up a song of repentance on behalf of a world that has sometimes neglected its obvious duties to doctors, and that they might show the reality of their penitence by a generous donation to Epsom College, and more especially to its projected "Pension Fund" for aged doctors and their widows. In order that they may take immediate advantage of the gracious mood which we cannot doubt they are now experiencing, we venture to inform them that the Secretary of the Institution is Mr. R. Freeman, and that the office is at 37, Soho Square.

